# Association between magnetic field exposure and miscarriage risk is not supported by the data

**DOI:** 10.1038/s41598-021-01391-3

**Published:** 2021-11-12

**Authors:** David Robert Grimes, James Heathers

**Affiliations:** 1 School of Physical Sciences, Dublin City University, Dublin, Ireland; 2 Department of Oncology, University of Oxford, Oxfordshire, Oxford, UK; 3 Cipher Skin, Denver, CO USA

**Keywords:** Epidemiology, Biophysics

**arising from**: D-K. Li et al.; *Scientific Reports* 10.1038/s41598-017-16623-8 (2017).

## Introduction

In “Exposure to Magnetic Field Non-Ionizing Radiation and the Risk of Miscarriage: A Prospective Cohort Study”, De-Kun Li and colleagues purport to find strong association between Magnetic Field (MF) exposure and prevalence of miscarriage in women with “high” MF exposure. From a large sample of women (n = 913), MF exposure was passively measured for 24 h, and the 99th percentile of those individual scores was treated as each woman’s ‘exposure index’. Within the indexes, any score over 2.5 milligauss (mG) was classified as “high” exposure, a cut-off reported as the 25th percentile of the MF indices. The experimenters then atrophied the groups, removing participants who did not deem their recorded day to be ’typical’ of their usual exposure. With just over half the subjects eliminated after self-reporting atypical recording days (n = 453), a comparison between low and high exposure groups (< 2.5 mG vs $\ge$ 2.5 mG) suggested an increase in miscarriage rate in the high exposure group, but—crucially—no dose-response effect was found. From this, the authors concluded there was a strong association between MF exposure and miscarriage risk, a finding that was widely publicised. While we commend the authors for their commitment to investigating such effects, our opinion is that this work exemplifies a number of deeply unsound methodological choices that nullify its strong conclusion, which we will elucidate here, with subsequent discussion on how such missteps can be avoided by all researchers.

## Methodological and statistical issues

### Anomalous attrition of sample

The ad hoc choice to jettison over half the initial sample is a methodological decision that drives all the resultant conclusions. The ‘Typical Day’ exposure grouping the authors report is extremely unusual, as per the following:

1. Just over half the sample did not experience a ‘typical’ day. In other words, it was more typical for participants analysed to have an ‘atypical’ day than a ‘typical’ one. This was in spite of the fact that participants were presumably instructed to ensure their monitoring day was reflective of their normal routine to participate in a monitoring study of daily life.
2. ‘Typical’ vs. ‘non-typical’ status was decided by interview. The authors assert this analytical distinction is necessary, citing one of their previous publications which did not find any association between MF and miscarriage risk, but noted removing the group deemed atypical seemed to nudge the results towards significance. Such post hoc analysis could easily lead to data-dredging, as there is no a priori reason why atypical days should have lower net exposure than typical days. While the authors insist this distinction is vital, they did not employ it subsequently, even after the collection of very similar tranches of data^1,2^.

While it is possible, technically, that the above is simply the features of an extremely idiosyncratic experimental and analytical combination, the following is not: the attrition of the sample varies significantly in one particular subgroup, as can be seen in Table 1. A priori, the selection process between the typical and atypical day cohorts should be entirely random—there should be no reason any one sub-category should be more or less likely to be excluded, and one would expect all groups to be reduced by approximately 50$\%$. This does indeed happen for all groups except the highlighted group where attrition is far more pronounced. This is critical, as comparisons with this group (‘Miscarriages in the lowest exposure quartile’) drives all subsequent conclusions.

**Table 1:** Data from paper (with attrition rate calculated).

Subject grouping	Original number	Typical day sample	Attrition rate (%)
Lowest quartile (no miscarriage)	183	95	48.1
Lowest quartile (miscarriage)	36	11	69.4
Higher quartiles (no miscarriage)	530	263	50.4
Higher quartiles (miscarriage)	164	84	48.8

**Figure 1:**
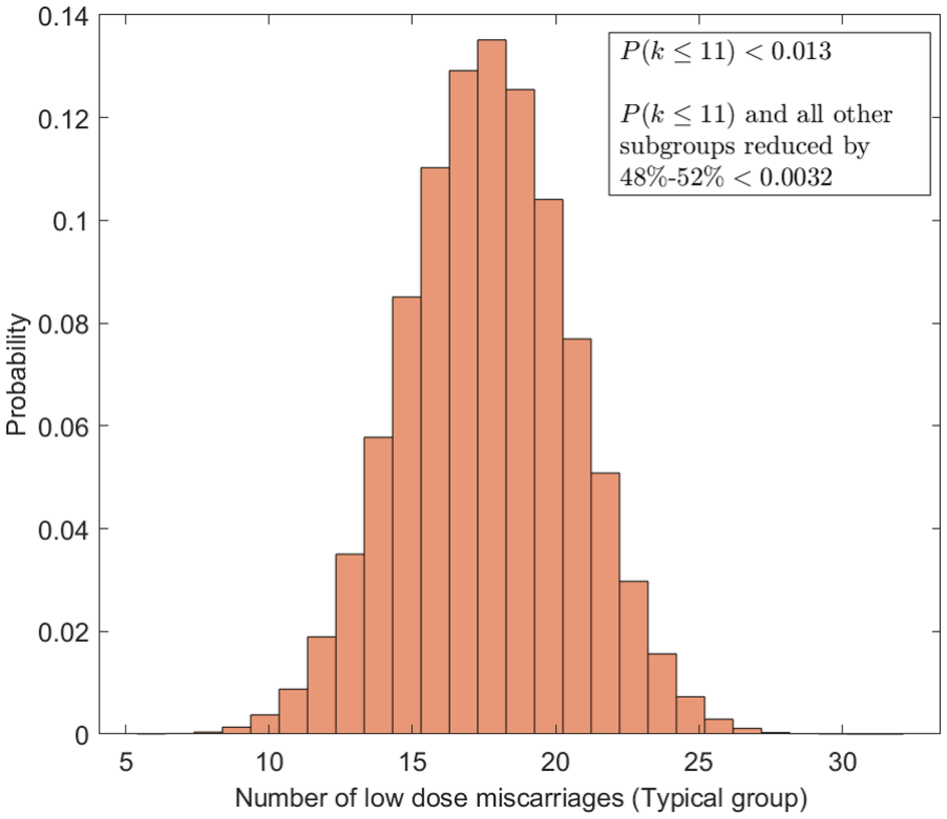
Simulation of the separation process into “typical day” for lowest quartile miscarriage. Level of attrition in this subgroup is significantly more than would be expected by chance. See text for details.

Because this low dose miscarriage group suffers more severe attrition than the other subgroups, and because this group also drives the seemingly strong conclusion of the paper, it is important to gauge how likely this configuration is to occur by chance. To investigate this, we simulated a random selection process of the 913 subjects down to 453, and ran the simulation 500,000 times. A histogram of results from this simulation is shown in Fig. 1. The modal number of lowest quartile miscarriages in the simulation is 18 (half of 36) as would be expected. The probability of this cohort having 11 or fewer participants is low ($p < 0.013$). When this occurs, it also tends to skew other groups in the analysis. Simulation results suggest that a scenario where the lowest quartile ‘no miscarriage’ group has 11 or fewer members AND the other groups are reduced by $50\% \pm 2\%$ is an exceedingly rare occurrence by chance alone ($p < 0.0032$). Taken together, this suggests something remiss in the data selection process. It further suggests that this group should not be used as a reference point, as their sub-selection from an initially balanced cohort cannot be justified as representative. This is unfortunate, as no comment is made as to this extreme level of attrition and all subsequent analysis is conducted as if it were (see Tables 2,3,4 in original paper). This attrition is not well-justified, and undermines the statistical power of the trial.

### Unstable quartiles

**Figure 2:**
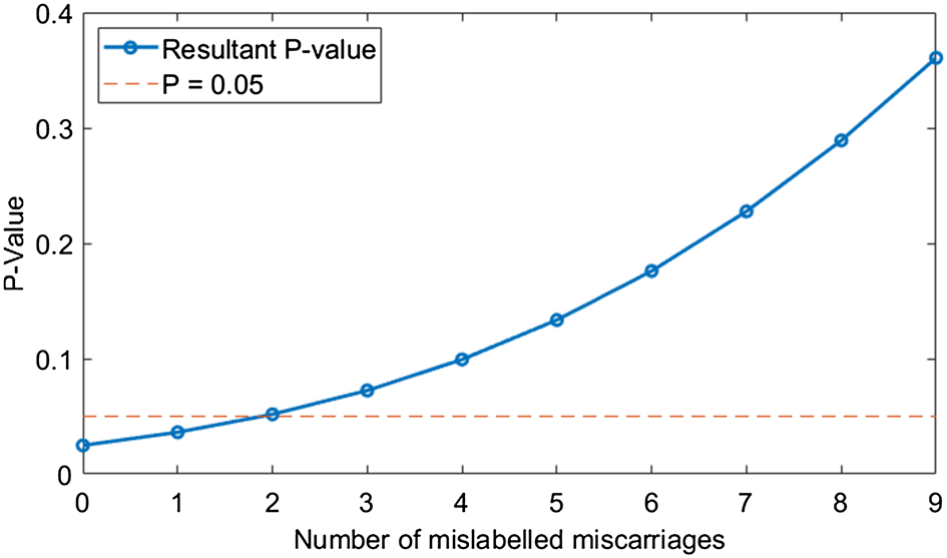
A discrepancy of 9 women in the full sample can mean anything from 0–9 mislabelled miscarriages, with strong implications for the ostensible significance of the result. Dotted line depicts $\alpha = 0.05$ significance level.

There is a further issue in terms of both the reported data and how the analysis was designed. Returning to Table 1, a first glance at both the full and typical day subset suggests results appear significant for both($\chi ^2$ test for all participants, $p=0.0249$, $\chi ^2$ test for typical day subgroup $p=0.0022$), with non-significant results for non-typical days. But there is problem with the data reported—by definition, *the lowest quartile group should contain a quartile of data* (in this case, of the MF index data, linked to each patient). Likewise, the comparison group should contain three-quarters of all participants. Instead, the lowest quartile (before attrition) contains 219 subjects when it should contain approximately 228, a discrepancy of about 9 women. These cases are incorrectly coded, and this greatly affects the significance of the result, as illustrated in Fig. 2. As already outlined, the typical day selection is unusual and even quartiles should not be expected after attrition. It can however be demonstrated that mislabelling of even 2 miscarriages in this set can negate the seemingly strongly significant signal, which is problematic where mislabelling of at least 9 cases has certainly occurred.

In response to this, the authors clarified that erroneous quartiles were due to round-off error, based on reduced a three decimal place value for first quartile (2.525 mG) to one place of decimals (2.5 mG). This may explain the genesis of the incorrect quartile value, as similar issues appear in other papers on the topic by the authors; a 2011 paper exploring a link between MF and asthma has the lowest 10th percentile containing 19 excess participants ($\approx 13\%$)^3^, while a 2020 paper seeking a link between MF and ADHD was retracted for similar reasons^2^. While the source of this error is clear, it does not circumvent the issue this quantification error introduces.

### Inappropriate dichotomisation and thresholding of data

All the data gathered in the original paper and the author’s associated works are continuous, and should be analysed as such. There are an array of methodological reasons why it is inappropriate to force a dichotomy on a continuous data set^4–7^, and the author’s arbitrary decision to impose a high/low cut-off at the first quartile confounds further analysis. By contrast, if data had been reported continuously, a clear relationship between miscarriage rates and MF exposure would have been far easier to observe if it existed. The author’s response was that the dichotomy between high and low was created because the exposure threshold for adverse effects was unknown. However, this response is unsatisfactory because (a) dose-relationship effects would be more apparent and testable with the continuous, non-dichotomised data available, and (b) dichotomising data without valid reason to do so is not good practice.

**Figure 3:**
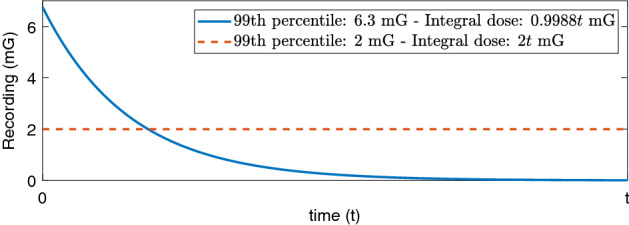
The blue curve has a 99th percentile of 6.3 mG, rendering it a highest quartile MF index by Li et al’s schema. The red curve would be in the lowest quartile with a 99th percentile of only 2 mG, yet the woman whose exposures are recorded on the red curve would receive more than double the total dose of the participant whose exposure follows the blue curve.

The author’s technique for ascertaining harm threshold is also suspect. With 913 women wearing an ’EM meter’ for 24 h, recording at 10 s intervals, one would expect approximately 8640 measurements recorded per woman. The authors compressed this rich data to a single metric, assinging the 99th percentile of these recordings as a woman’s MF index. There is little evidence for this, bar a reference to a previous paper by the same author group, which did not find an association between miscarriage risk and average magnetic field level. The authors found however that arbitrarily stratifying subjects in deciles by maximum level of a 24 h MF exposure suggested a higher risk in some cohorts, although the dose-response curve was non-monotonic and the $95\%$ confidence intervals of relative risk crossed unity in several instances^8^. The problem is that this is a loaded inference which could readily lead to selection of false positives^9–11^. There is no clear biophysical rationale for this metric, which differs even from that used in the author’s related works. Nor were other reasonable exposure metrics considered; the integral of a continuous dose can be very different to its extreme percentiles, as illustrated in Fig. 3. As the authors had continuous data available, arbitrarily selecting a questionable threshold without solid biophysical rationale is suspect without further justification, outlined in the discussion section.

## Biophysical issues

A repeated problem in the work is the conflation between different physical phenomena, particularly magnetic fields and non-ionizing radiation. Magnetic fields are not a form of radiation under any accepted conventional definition of the term, yet the central thesis of the paper conflates this with non-ionizing radiation several times. This is patently false—non-ionizing radiation refers to photons in the electromagnetic spectrum below a threshold frequency, lacking the requisite energy per quantum to eject an electron from an atom or molecule. The ionization threshold is usually given conservatively as 10 eV, with light below this threshold (including visible, infrared, radio-frequency, and microwave radiation) deemed non-ionizing. Magnetic fields are not a form of radiation, so invoking them in this context is, again, misleading. While it is true that electromagnetic (EM) fields are comprised of oscillating electric and magnetic fields, in light-matter interactions, electric fields utterly dominate and magnetic effects are negligible^12^.

The issue of the above inappropriate conflation leads citations and inferences which simply do not apply. Some examples: the authors refer to a National Toxicology Programme (NTP) study which they claim* “..found that the cancer risk due to MF exposure observed in their experimental animals matched the cancer cell types that had been reported in previous epidemiologic studies in human populations”*. This is patently untrue—the NTP study did not look at MF at all, focusing instead on exposure to radio-frequency EM radiation. In addition, its central conclusion has been roundly criticised for questionable methodology^13^. The sample conflation error occurs again when the author’s state “*the International Agency for Research on Cancer (IARC) has classified MF as a possible carcinogen*”—again, this is false. The IACR designated radio-frequency radiation as a group 2B agent, a designation frequently misunderstood as implying evidence of harm. This interpretation is however incorrect, as reiterated in the most recent IARC communication^14^.

*Even if* one could treat the magnetic fields considered in the author’s work as EM radiation, it would not circumvent another serious issue within this work. The frequency range of magnetic fields recorded in the study was 40–1000 Hz. But Wi-Fi, cellular, and mobile frequencies operate in the radio-frequency spectrum, typically at frequencies of 2–6 GHz^15^—a factor of> 2-million fold greater the MF field frequencies detected in this work. It is biophysically implausible to treat such different frequencies as equivalent, even if the conflation between MF and EM could be justified, which it cannot be. More strikingly, the authors do not offer any convincing reason why they might expect these relatively small exposures fixated upon in the paper to be harmful, nor do they offer proper context for these claims. The Earth’s magnetic field, for example, varies across the Earth from 250–650 mG^16^—roughly 100–260 fold the threshold for provoking increased miscarriage that the authors suggest. In response to this query, the authors posited that this can be disregarded as the Earth’s magnetic field is static, but this is not true at the mG scale considered. Not only is the Earth’s magnetic field constantly perturbed by geomagnetic events^17^, secular variation^18^, and other daily influences^19^, but also displays significant geographical variation. The variation suggested as dangerous in this work is orders of magnitude below typical variation of the Earth’s magnetic field strength, and this is not sufficiently justified in the text or theory discussed.

## Discussion

It is critical that health effects of new and emergent technologies are carefully monitored, and that the thresholds of safety are updated as the evidence base grows. Yet based on the issues raised here, we feel the conclusions of Li et al’s paper are not justified by the data reported. It is not our intention to criticise the researchers on any personal level, as we feel such investigations are vital. However, for public trust in science to be maintained, strong scrutiny into matter of strong harms caused by common exposures is demanded. With topics of public contention, methodologically unsound work has the potential to do severe harm, even if conducted with good, peripheral, or unwitting intentions. In this instance, we only became aware of this paper after we were contacted by major international media outlet to comment on it. The contacting producers relate that they came across this work on a digital anti-5G platform, where it was shared as “proof” of the irreparable harms of radiofrequency. This is not the fault of the authors, of course, and it is far from a new experience for researchers to be frustrated with inaccurate or out-of-context interpretations of their published work^20^, but a lay-reading of the conclusions of Li et al. would likely leave an impression of a clear link between radiofrequency radiation and miscarriage.

It is also important to point out that the methodological and statistical issues raised herein afflict a great deal of published science and medicine, and one of our motivations in outlining our concerns was to illuminate why certain research practices can readily give rise to misleading results. Issues raised here are broadly reflective of many past debates within the life and social sciences—dichotomising a continuum of measurement (CITES), setting a category of interest after inspection of the data, the dangers of ’over-correcting’ outlier or unusual data according to decision rules related to an expected distribution^21^, the loss of power within subgroup analysis^22,23^ and so on. In short, the criticisms contained here are not unusual, nor particularly novel, but are couched within a long intellectual tradition of scientists improving each others methodological rigour over time.

## References

[1] Su X-J (2014). Correlation between exposure to magnetic fields and embryonic development in the first trimester. PLoS One.

[2] Li D-K (2020). Notice of retraction and replacement li et al association between maternal exposure to magnetic field nonionizing radiation during pregnancy and risk of attention-deficit/hyperactivity disorder in offspring in a longitudinal birth cohort. JAMA Netw. Open..

[3] Li D-K, Chen H, Odouli R (2011). Maternal exposure to magnetic fields during pregnancy in relation to the risk of asthma in offspring. Arch. Pediatr. Adolesc. Med..

[4] Altman DG, Royston P (2006). The cost of dichotomising continuous variables. Br. Med. J..

[5] Irwin JR, McClelland GH (2003). Negative consequences of dichotomizing continuous predictor variables. J. Market. Res..

[6] MacCallum RC, Zhang S, Preacher KJ, Rucker DD (2002). On the practice of dichotomization of quantitative variables. Psychol. Methods.

[7] Nuzzo RL (2019). Making continuous measurements into dichotomous variables. PM&R.

[8] Li D-K (2002). A population-based prospective cohort study of personal exposure to magnetic fields during pregnancy and the risk of miscarriage. Epidemiology.

[9] Ioannidis JP (2005). Why most published research findings are false. PLoS Med..

[10] Colquhoun D (2014). An investigation of the false discovery rate and the misinterpretation of p-values. R. Soc. Open Sci..

[11] Grimes DR, Bauch CT, Ioannidis JP (2018). Modelling science trustworthiness under publish or perish pressure. R. Soc. Open Sci..

[12] Burresi M (2009). Probing the magnetic field of light at optical frequencies. Science.

[13] International Commission on Non-Ionizing Radiation Protection and others (2020). Icnirp note: Critical evaluation of two radiofrequency electromagnetic field animal carcinogenicity studies published in 2018. Health Phys..

[14] Wild, C. P., Weiderpass, E. & Stewart, B. W. editors. World Cancer Report: Cancer Research for Cancer Prevention. Lyon, France: International Agency for Research on Cancer. Available from: http://publications.iarc.fr/586 (2020). 39432694

[15] Grimes DR, Bishop DV (2018). Distinguishing polemic from commentary in science: Some guidelines illustrated with the case of sage and burgio (2017). Child Dev..

[16] Finlay CC (2010). International geomagnetic reference field: the eleventh generation. Geophys. J. Int..

[17] Fleming J, Harradon H, Joyce J (1939). Seventh general assembly of the association of terrestrial magnetism and electricity at Washington, DC, September 4–15, 1939. Terr. Magn. Atmos. Electr..

[18] Jackson A, Jonkers AR, Walker MR (2000). Four centuries of geomagnetic secular variation from historical records.. Philos. Trans. R. Soc. Lond. Ser. A Math. Phys. Eng. Sci..

[19] Davies CJ, Constable CG (2020). Rapid geomagnetic changes inferred from earth observations and numerical simulations. Nat. Commun..

[20] Grimes DR (2021). Medical disinformation and the unviable nature of covid-19 conspiracy theories. PLoS One.

[21] Miller J (1991). Reaction time analysis with outlier exclusion: Bias varies with sample size. Q. J. Exp. Psychol..

[22] Guillemin F (2007). Primer: The fallacy of subgroup analysis. Nat. Clin. Pract. Rheumatol..

[23] Tanniou J, Van Der Tweel I, Teerenstra S, Roes KC (2016). Subgroup analyses in confirmatory clinical trials: Time to be specific about their purposes. BMC Med. Res. Methodol..

